# Implant-associated biofilms of *Staphylococcus aureus* and *Enterococcus faecalis* clinical isolates on expanded polytetrafluoroethylene suture in *Galleria mellonella* model

**DOI:** 10.1038/s41598-025-26971-5

**Published:** 2025-11-12

**Authors:** Kamran Ahmad Mirza, Sandor Nietzsche, Tinatini Tchatchiashvili, Oliwia Makarewicz, Mathias W. Pletz, Lara Thieme

**Affiliations:** 1 Institute of Infectious Diseases and Infection Control, Jena University Hospital, Friedrich-Schiller-University Jena, Am Klinikum 1, 07747 Jena, Germany; 2 Leibniz Center for Photonics in Infection Research, 07747 Jena, Germany; 3 Research Training Group 2723 ‘Materials-Microbes-Microenvironments’ (M- M-M), Jena University Hospital, Friedrich Schiller University, 07747 Jena, Germany; 4 Center for Electron Microscope, Jena University Hospital, Friedrich-Schiller- University Jena, 07743 Jena, Germany

**Keywords:** Antibiotic, Biofilm, Gram-positive, Vancomycin, Rifampicin, Invertebrate model, 3Rs, Diseases, Microbiology

## Abstract

Implant-associated biofilm infections, particularly those caused by *Staphylococcus aureus* and *Enterococcus faecalis*, present significant challenges in clinical settings, often necessitating surgical removal. This study investigates the potential of the invertebrate model *Galleria mellonella* for evaluating biofilm formation of *S. aureus* and *E. faecalis* clinical isolates, the leading causes of implant-associated infections. Utilizing expanded polytetrafluoroethylene (ePTFE) sutures as a surrogate for cardiac implants, we employed two biofilm formation methodologies reflecting the two main routes of implant infections: *in vivo* biofilm formation within larvae mimicking pathogen spread and pre-formed biofilm transplantation. Scanning electron microscopy revealed complex biofilm structures for the biofilm formed inside of the larvae on implant, closely mimicking clinical implant biofilm. Antibiotic combination of vancomycin and rifampicin demonstrated mean 5 log_10_ reductions in bacterial CFU count per larva and 50% improved survival, proving highly effective. The study highlights the *G. mellonella* model’s potential for preclinical biofilm research, offering a cost-effective and ethical alternative to vertebrate models while providing valuable insights into biofilm-related infections and their treatment.

**Supplementary Information:**

The online version contains supplementary material available at 10.1038/s41598-025-26971-5.

## Introduction

Implant-associated biofilm infections present a significant challenge in medicine. Despite the combination of surgical debridement and antibiotic therapy, 30–40% of cases result in treatment failure and necessitating invasive procedures like implant removal^1^. *Staphylococcus aureus* and *Enterococcus faecalis* are the primary pathogens implicated in these infections, with *S. aureus* being the leading pathogen in prosthetic valve endocarditis^2,3^. Both pathogens are well-known biofilm formers, contributing to the persistence of infective endocarditis on native and prosthetic valves and resistance to treatment^4,5^. As these infections persist, a more comprehensive understanding of biofilm formation on implants is crucial for developing effective strategies to overcome this enduring challenge.

The use of vertebrate models in studying implant-associated infections is limited by ethical concerns, particularly in experiments involving biofilm formation and treatment^6^. This is due to the prolonged observation and invasive procedures, significant stress, pain, and potential suffering for the animals involved. In response to these concerns, the 3R principles (replacement, reduction, refinement) have driven the search for alternative models^7^. The greater wax moth larvae, *Galleria mellonella*, have emerged as a promising, cost-effective, and ethically favourable model for studying human bacterial pathogens^8,9^. With an innate immune system similar to mammals and a proven susceptibility to diverse human pathogens, *G. mellonella* provides clinically relevant insights into implant-associated biofilm formation and treatment strategies.

The study investigates implant-associated biofilm formation of three distinct clinical isolates of *S. aureus* and *E. faecalis* in the *G. mellonella* model. We introduced an expanded polytetrafluoroethylene (ePTFE) suture as a surrogate implant, mimicking prosthetic valve endocarditis pathogenesis, where biofilm formation starts on the suture material at the tissue-implant interface^10^. This research presents a comparative analysis of two biofilm formation methodologies: (i) Implant-in-Larvae (IL) — a model where sterile ePTFE sutures were implanted into larvae, followed by infection with planktonic bacteria after 1 h. This approach simulates biofilm formation post-placement of an implant, due to pathogen spread. (ii) Pre-formed biofilm (PF) — a model where ePTFE implants were pre-incubated with bacterial suspensions in vitro to allow biofilm development, before being implanted into larvae. This approach reproduces clinical scenarios in which medical devices or surgical materials become contaminated before implantation or during implantation, as well as cases of prosthetic re-implantation or revision where pre-colonized materials are introduced into the host. The bacteria quantification was performed via CFU count while scanning electron microscopy (SEM) was employed to visualize the biofilm structure and complexity. Finally, we assess the model’s efficacy in evaluating antimicrobial activity against bacterial biofilm by applying the recommended antibiotic regimen of vancomycin and rifampicin combination^11^ for *S. aureus* prosthetic endocarditis, examining its impact on biofilm formation and eradication.

## Results

### *G. mellonella* and systemic infection

**Fig. 1:**
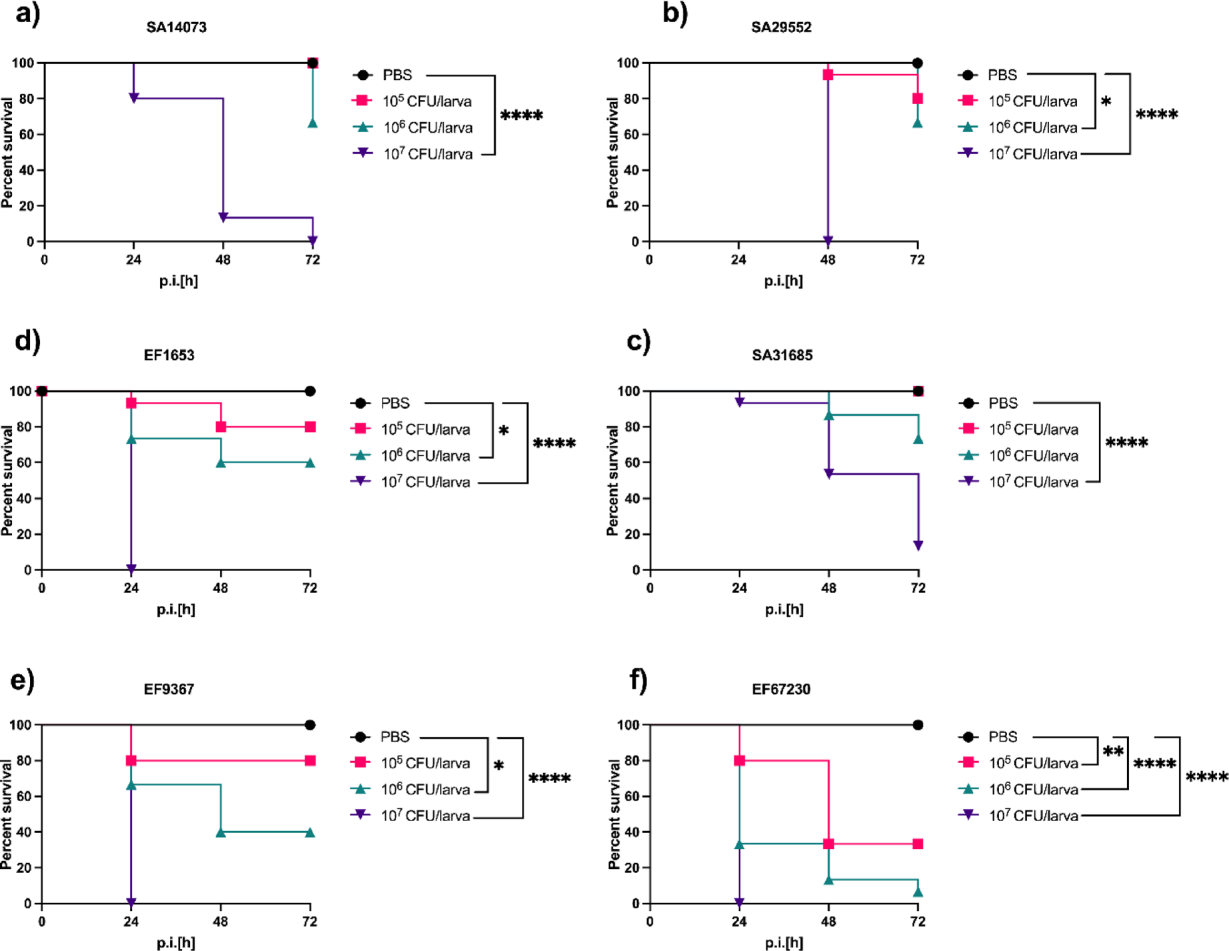
Survival curves of the larvae infected with different dosage of *S. aureus* isolates SA14073 (**a**), SA29552 (**b**) and SA31685 (**c**) and *E. faecalis* isolates EF1653 (**d**), EF9367 (**e**) and EF67230 (**f**). The larvae were infected with different bacterial dosages via proleg, followed by a 72 h incubation period, with survival assessment conducted every 24 h. The statistical significances were analysed using Log-Rank test and *p* < 0.05 was considered significant.

The larvae were injected with bacterial isolates to evaluate the bacterial virulence toward larvae. Upon injection, the larvae’s immune response is characterized by pathogen encapsulation and melanin production, which manifests as visible darkening of the larvae^12^. This pigmentation change served as a practical indicator to assess the impact of varying bacterial dosages on larval health. Therefore, larvae were administered a series of 10-fold increasing bacterial dosages, followed by a 72 h incubation period, with survival monitored at 24 h intervals. An inoculum of 10^5^ CFU demonstrated a larval survival rate exceeding 80% after 72 h of infection (Fig. 1a–c). Conversely, higher bacterial dosages, such as 10^7^ CFU, resulted in 100% larval mortality (Fig. 1a). Additionally, with an inoculum of 10^6^ CFU, the larval survival rate remained below 80% (Fig. 1a,b). A similar pattern was observed in larvae infected with *E. faecalis* isolates (Supplementary data, Table S2), where increasing bacterial dosages corresponded with higher larval mortality rates (Fig. 1d,e). Notably, an exception was observed with *E. faecalis* isolate 67,230, where even a lower dosage of 10^5^ CFU led to a significant reduction in survival, with less than 50% of the larvae surviving after 72 h of infection, indicating the high virulence of this isolate (Fig. 1f).

### Implant biofilm in *G. mellonella*

**Fig. 2:**
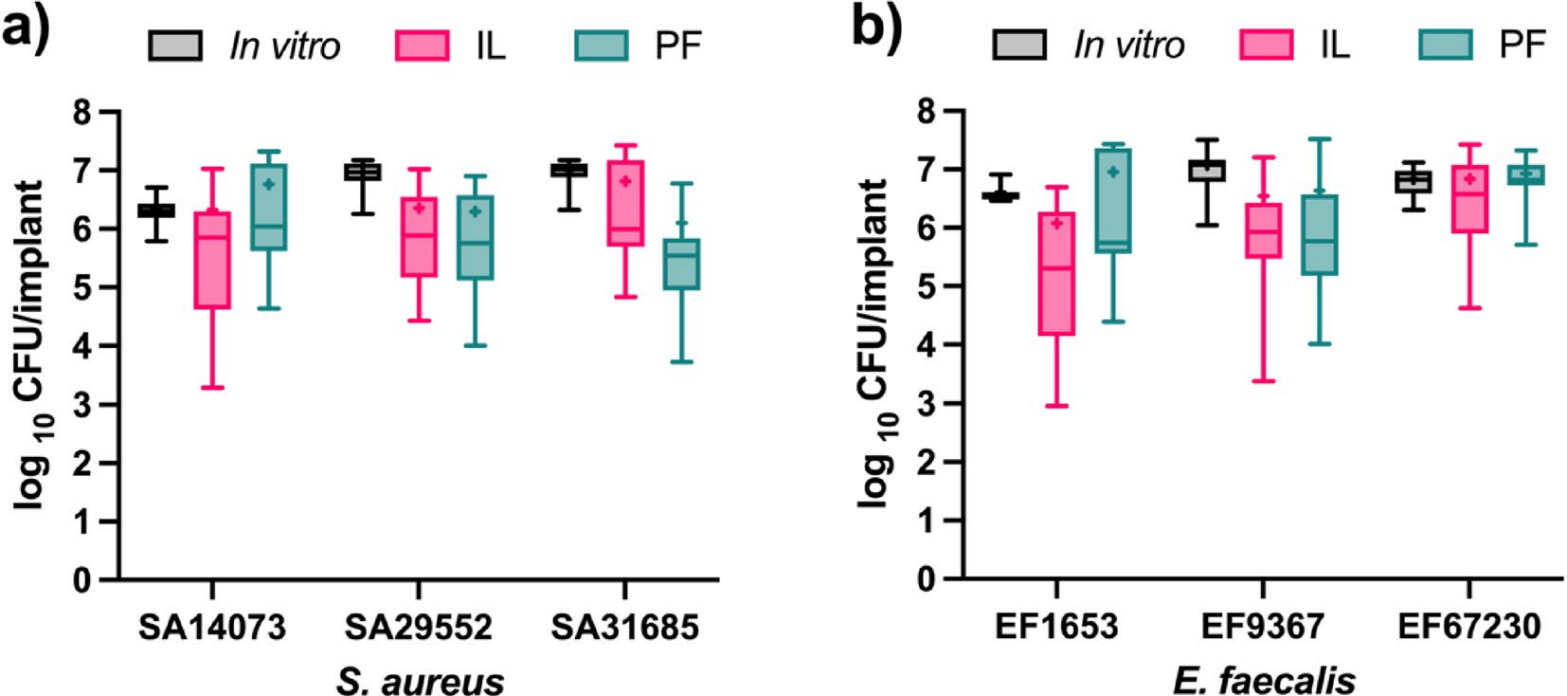
The CFU quantification of *S. aureus* isolates (**a**) and *E. faecalis* isolates (**b**) on the ePTFE implants. The data was represented as boxes and whiskers with 95th -5th percentiles, + represents mean and the line represents the median.

The ePTFE medical-grade material was used as the implant to assess and quantify bacterial biofilm formation under in vitro conditions. Quantification revealed bacterial loads ranging from approximately 6 to 7 log_10_ CFU per implant across all tested isolates (Fig. 2). Variability in CFU counts among strains of the same species was minimal; for example, the methicillin-sensitive *Staphylococcus aureus* (MSSA) isolate SA31685 (Supplementary data, Table S1) showed approximately 0.5 log_10_ higher CFU counts compared to the methicillin-resistant *S. aureus* (MRSA) isolate SA14073 on ePTFE. Both *S. aureus* and *E. faecalis* demonstrated substantial biofilm formation on the implants in the in vitro setting. Furthermore, CFU quantification of implants retrieved from larvae revealed comparable bacterial loads, with mean values ranging from 6 to 7 log_10_ CFU/implant for both the IL and PF methodologies (Fig. 2a,b). Despite different growth conditions, IL and PF implants showed similar CFU. In PF implants, host immune factors likely reduced viable counts after implantation, whereas IL biofilms developed under continuous immune pressure, balancing bacterial expansion. This consistency between in vitro and in vivo CFU counts highlights the reproducibility and robustness of the quantification methods across experimental settings.

**Fig. 3:**
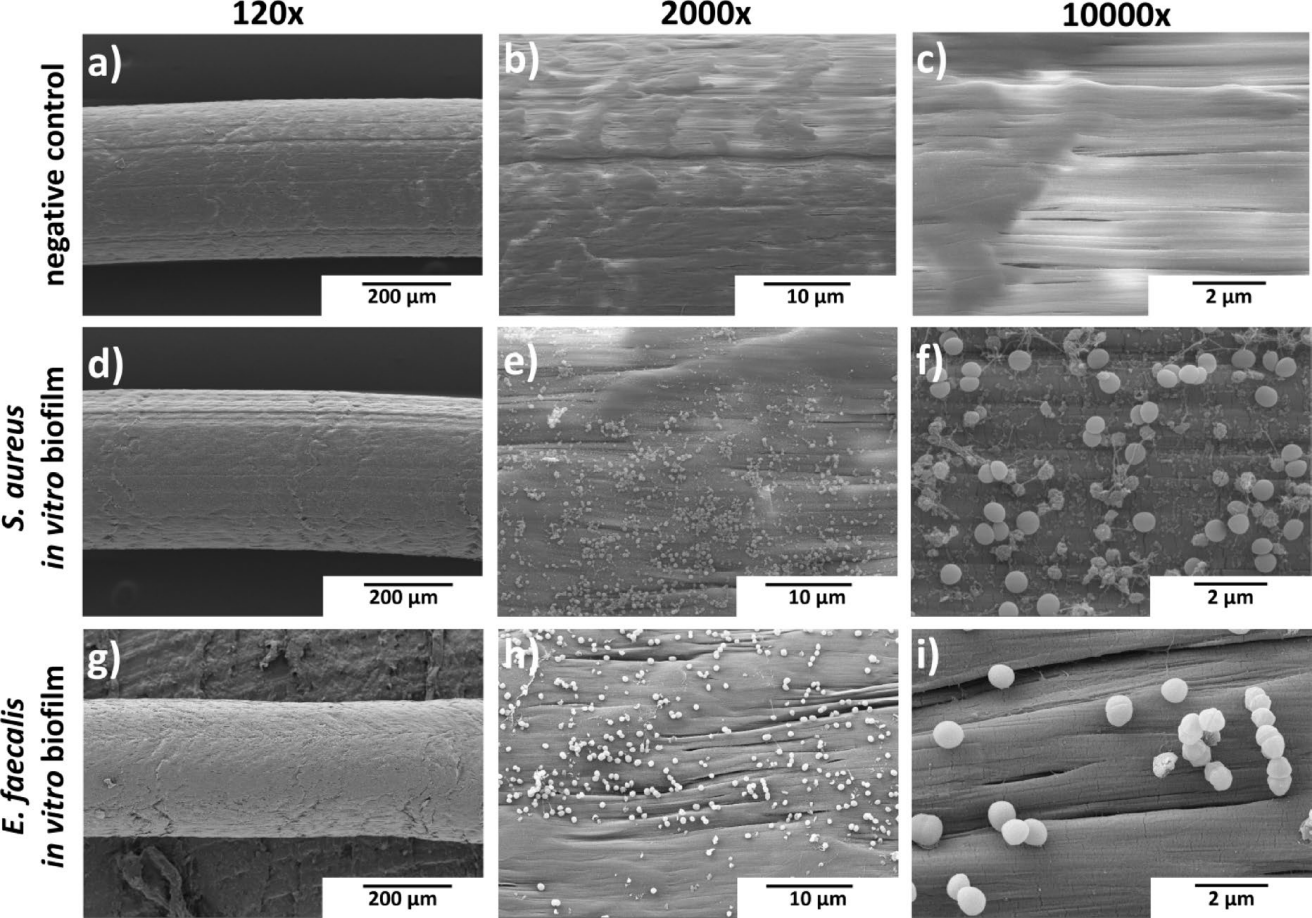
SEM images of ePTFE implant without bacterial attachment (**a**–**c**) and in vitro colonisation by *S. aureus* isolate SA14073 (**d**–**f**) and *E. faecalis* isolate EF1653 (**g**–**i**) for 48 h. The images showed different magnifications as indicated. The scale bar (black) represents 200 μm (a, d, g), 10 μm (b, e, h), and 2 μm (c, f, i).

SEM was used to visualize the structural features of in vitro biofilms formed by *S. aureus* and *E. faecalis* on ePTFE implants. Both species demonstrated initial stages of biofilm development on the ePTFE surface (Fig. 3). SEM images of *S. aureus* isolate SA14073 revealed small bacterial aggregates with limited extracellular matrix, indicative of early attachment and microcolony formation (Fig. 3d–f). In contrast, *E. faecalis* isolate EF1653 exhibited more uniform coverage of the implant, with formation of sheet-like bacterial layers and discrete microcolonies (Fig. 3g–i). These structural patterns are consistent with early-phase biofilm development and reflect species-specific differences in biofilm architecture under in vitro conditions. Sterile control implants showed no bacterial attachment (Fig. 3a–c).

**Fig. 4:**
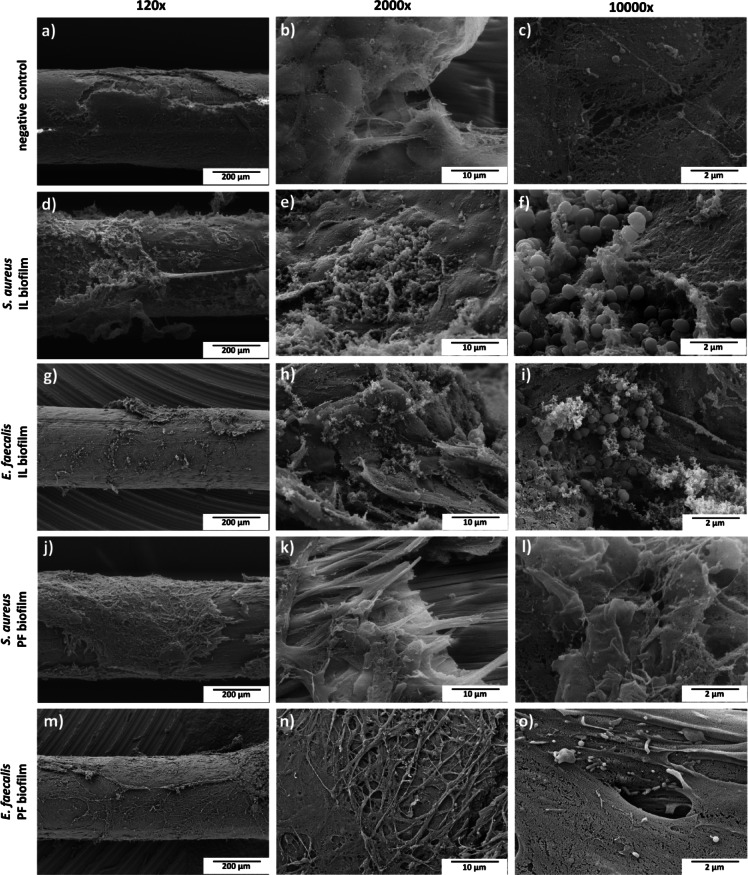
SEM comparison of IL and PF biofilm formation methods. Panels (**a**–**c**) display the SEM images of sterile ePTFE implants. Panels (**d**–**f**) and (**g**–**i**) illustrate biofilm formation by *S. aureus* isolate SA14073 and *E. faecalis* EF1653, respectively, using the IL method within *G. mellonella* larvae, where biofilm formation is facilitated by larval body materials. Panels (**j**–**l**) and (**m**–**o**) show the SEM images of SA14073 and EF 1653, respectively using the PF method, indicating a noticeable absence of biofilm formation. The scale bar (black) represents 200 μm (a, d, g, j, m), 10 μm (b, e, h, k, n) and 2 μm (c, f, i, l, o).

In IL model, both *S. aureus* isolate SA14073 and *E. faecalis* isolate EF1653 formed mature biofilms. SEM images showed dense bacterial aggregates embedded in an extracellular polymeric matrix, often associated with larval-derived material coating the implant surface (Fig. 4d–i). These observations reflect advanced biofilm development and suggest a facilitating role of the host environment in biofilm maturation and matrix composition. In contrast, SEM analysis of implants from the PF biofilm group revealed no visible bacterial clusters or classic biofilm structures. However, the entire surface of the implants was covered by a dense layer of host-derived material (Fig. 4j–o,m–o), which may have masked the underlying bacterial cells.

**Fig. 5:**
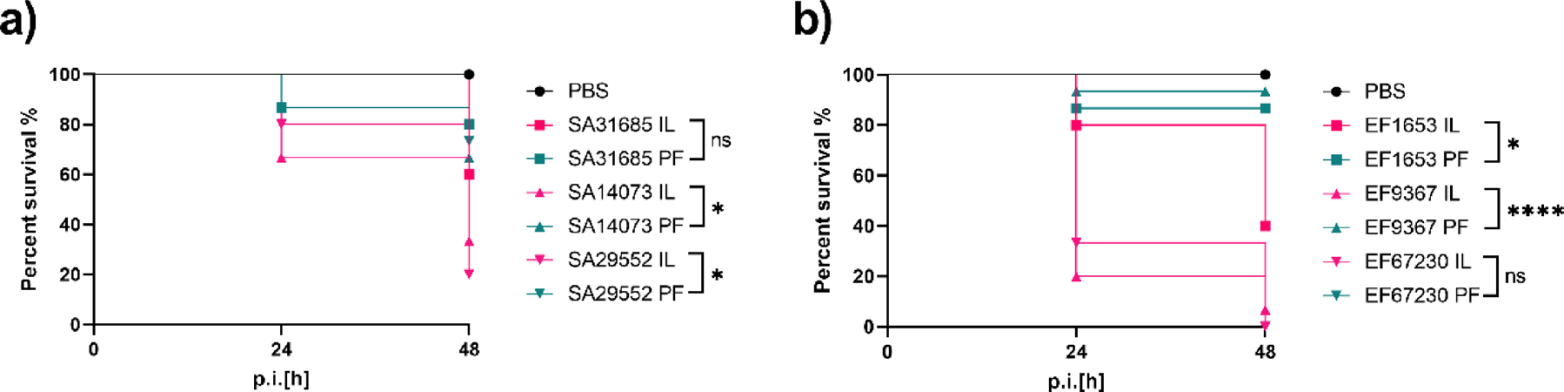
Survival of the larvae infected with implant biofilm of different methodologies IL and PF of *S. aureus* isolates (**a**) and *E. faecalis* isolates (**b**). Two distinct approaches were employed: IL biofilm, where the biofilm developed on the implant inside the larvae; and PF biofilm, involving biofilm formation on the implant in vitro prior to transplantation into the larvae. The larvae were incubated for 48 h and every 24 h survival was observed. The statistical differences were analyzed using Log-rank test and *p* < 0.05 was considered significant.

Larval survival was assessed to evaluate the impact of biofilm formation method on host mortality. Across all tested isolates, the IL method resulted in significantly higher mortality compared to the PF method (Fig. 5a,b). For instance, larvae infected with *S. aureus* isolate SA29552 exhibited a survival rate of only 33% in the IL group, whereas survival increased to 70% with the PF method. Similarly, for *E. faecalis* isolate EF9367, IL biofilms led to a survival rate of 8%, in contrast to 92% survival in the PF group. These differences were statistically significant (*p* < 0.05, log-rank test), indicating that biofilms formed in vivo within the IL were more detrimental to larval viability than PF biofilms.

### Assessment of antimicrobial effects in *G. mellonella*

To evaluate the efficacy of standard antimicrobial therapy against implant-associated biofilms, larvae infected via IL or PF methodology were treated with vancomycin, rifampicin, or their combination. In the IL model, treatment with rifampicin alone or in combination with vancomycin significantly improved larval survival. For instance, SA14073 IL biofilm-infected larvae showed a 100% survival rate with combination therapy, 90% with rifampicin alone, and 50% in the untreated control group. Similar trends were observed with SA29552, where survival increased from 30% (mock) to 90% following combination treatment (Table 1).

In the PF model, survival outcomes showed less pronounced differences between treatment and control groups. Although CFU reduction was evident, especially with rifampicin and combination therapy, survival benefit was less consistent. For example, in SA29552 PF biofilms, survival increased from 60% (mock) to 100% with treatment, but no significant difference was observed for SA14073 and SA31685, which showed high baseline survival. This suggests that PF biofilms elicit a weaker systemic infection phenotype, limiting the ability to assess treatment efficacy using survival as an endpoint. This weaker systemic infection phenotype in PF implants likely reflects the limited interaction between pre-formed biofilms and host immune factors. Because PF implants were immediately encapsulated by host-derived material, bacterial dissemination into the hemolymph was restricted, leading to reduced virulence despite comparable CFU counts. In contrast, IL biofilms formed in direct interaction with the host environment, promoting stronger immune activation and higher larval mortality.

**Table 1:** Percent survival (%) of the larvae after 24 h of treatment against biofilms formed by *S. aureus* isolates applying the IL or PF method.

Bacterial species	Larvae percent survival (%) post 24 h of treatment							
	IL implant biofilm + Treatment				PF implant biofilm + Treatment			
Treatment	Mock (1 x PBS)	20 mg/L vanco	5 mg/L rifa	Abs. combination	Mock (1 x PBS)	20 mg/L vanco	5 mg/L rifa	Abs. combination
SA14073	50	70	90	100	70	100	100	100
SA29552	30	70	80	90	60	80	100	100
SA31685	80	75	88	88	85	100	100	100

### CFU quantification and SEM analysis

**Fig. 6:**
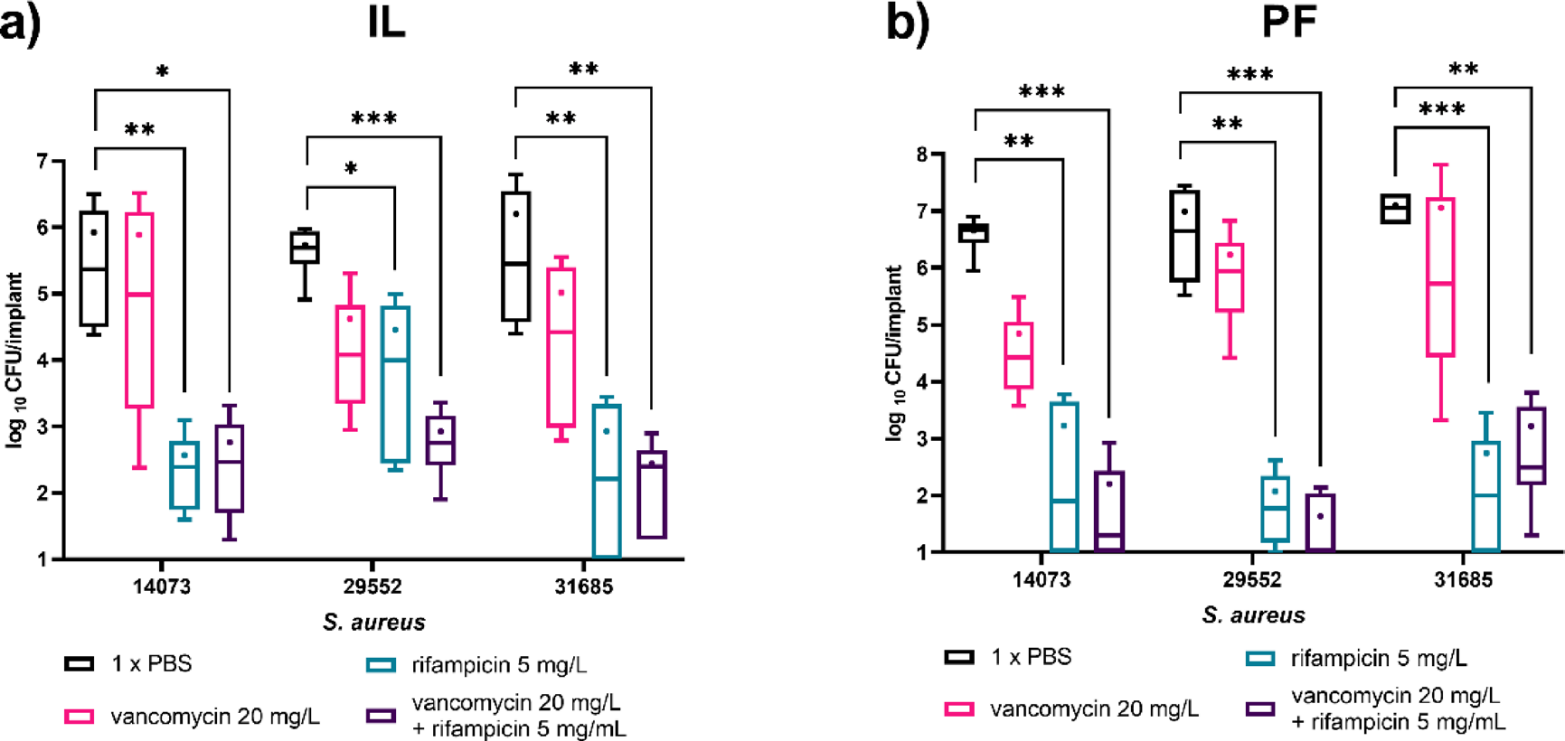
The log_10_ CFU reduction of bacterial biofilm of *S. aureus* isolates SA14073, SA29552 and SA31685 on the implanted material with IL (**a**) and PF (**b**) biofilm methodologies. The data was compared using Kruskal-Wallis test and *P* < 0.05 was considered significant. The data was represented as boxes and whiskers with min to max, the line represents the median and (+) represents the mean.

**Fig. 7:**
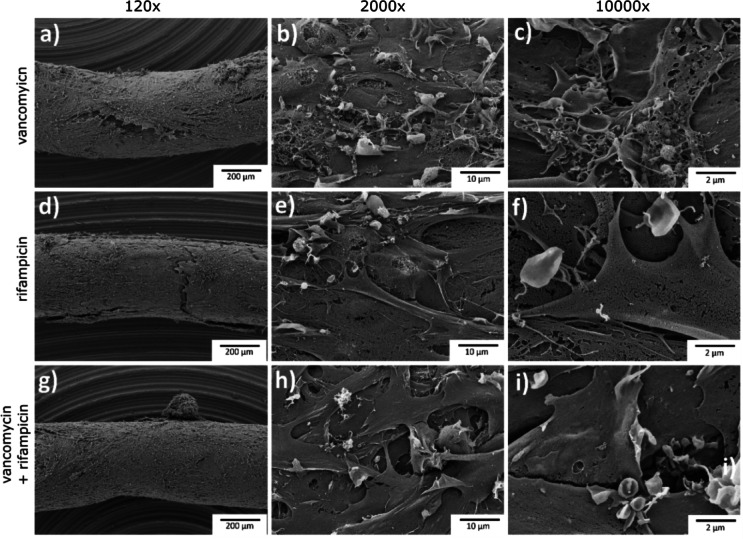
The post antibiotic treatment SEM analysis of *S. aureus* isolate SA14073 biofilms formed on implants with IL methodology. The biofilm treated with 20 mg/L vancomycin (**a**–**c**), 5 mg/L rifampicin (**d**–**f**) and combination of vancomycin and rifampicin (**g**–**i**). After 24 h of treatment, implants were taken out of the larvae, washed with 1 x PBS, fixed and SEM was performed. The images showed different magnifications as indicated on the panels. The scale bar (black) represents 200 μm (a, d, g), 10 μm (b, e, h) and 2 μm (c, f, i) magnification.

Quantitative CFU analysis supported the survival findings. In the IL model, combination treatment achieved up to 3 log_10_ CFU reduction, while rifampicin alone produced similar reductions across all isolates (Fig. 6a). In PF biofilms, both rifampicin and the combination achieved up to 5 log_10_ reductions in CFU (Fig. 6b). SEM imaging of treated IL and PF implants further confirmed bacterial clearance, showing disrupted or absent bacterial cells after rifampicin or combination therapy (Fig. 7) and (Supplementary data, Fig. 1). These results validate the IL model as a robust system for assessing antimicrobial activity against mature, host-associated biofilms.

## Discussion

Implant-associated infections caused by biofilm-forming pathogens such as *S. aureus* and *E. faecalis* remain a major clinical challenge, particularly in prosthetic valve endocarditis. The biofilm mode of growth confers enhanced tolerance to antibiotics and immune evasion, often resulting in treatment failure and the need for surgical intervention. In this study, we developed and characterized a *G. mellonella* implant model using clinically relevant ePTFE suture material to investigate biofilm formation and evaluate therapeutic strategies in a physiologically meaningful and ethically favorable system.

The survival of the larvae infected with different bacterial dosages aligned with the previous studies depicting the direct proportional relationship between bacterial load and larvae mortality^13^. The exception to the pathogenicity of the *E. faecalis* EF 67230 isolate can be due to genetic factors of the bacteria, as certain bacterial isolates have evolved mechanisms that enhance their pathogenicity towards certain hosts. For instance, a study utilizing the *G. mellonella* infection model with *E. faecalis* reported rapid larval death within 30 min post-infection with 10^5^ CFU, attributed to the activity of gelatinase, an enzyme that degrades inducible antimicrobial peptides in the larvae^14^.

Quantitative and structural analyses demonstrated consistent bacterial colonization of ePTFE implants under in vitro and in vivo conditions. CFU enumeration showed similar biofilm loads (~ 6–7 log_10_ CFU/implant) across all conditions, indicating the robustness and reproducibility of the setup. SEM imaging of in vitro biofilms confirmed early attachment, typical of initial biofilm development. In biofilm stages, the first step is initial attachment followed by microcolony formation in which bacteria begin to multiply and form small aggregates with small production of biofilm matrix^15^. The absence of intricate or complex biofilm may be attributed to the absence of complex environment such as in vivo conditions^16^. However, the IL methodology supported the formation of mature biofilm structures in vivo, as evidenced by dense bacterial aggregates embedded in extracellular matrix intertwined with larval tissue-derived material. These findings are consistent with previous studies demonstrating that in vivo host factors, including protein deposition and immune responses, critically shape biofilm architecture and maturation^16^. Conversely, PF biofilms, despite having similar bacterial CFU counts, were not visible by SEM and surrounded by host-derived material, adding to the limitation of SEM as it cannot visualize the encapsulated biofilm by host-derived material. The PF implants likely represent a more dormant or encapsulated infection state, resulting in reduced virulence and minimal impact on host survival^17^. Complementary methods with fluorescent labelling with Fluorescence Lifetime Imaging Tomography (FLIT) would allow direct visualization of bacterial presence and viability within both IL and PF implants and will be pursued in future work. Survival outcomes further highlighted the biological relevance of the IL model. Biofilms formed within the host led to significantly increased larval mortality compared to PF implants. This is likely due to the immune interaction during biofilm development in the IL group due to the bacterial injection in the hemolymph, which promoted more aggressive bacterial phenotypes and enhances host damage.

The model was further used to evaluate antimicrobial treatment efficacy. Rifampicin, alone or in combination with vancomycin, significantly reduced bacterial burden in both IL and PF biofilms, with reductions from 3 to 5 log_10_ CFU/implant. Rifampicin’s superior activity is likely due to its ability to penetrate biofilms and target dormant cells^18^, whereas vancomycin, due to its larger molecular size and mode of action, is less effective against biofilm-embedded or non-growing bacteria^19^. Therefore, the comparable CFU reductions in IL and PF implants mainly reflect rifampicin efficacy rather than equal antibiotic penetration across treatments. Importantly, the IL model allowed discrimination of treatment efficacy in terms of both bacterial reduction and survival benefit—metrics not consistently assessable in the PF model due to limited host damage and low baseline mortality. Our study did not assess post-treatment resistance development, which remains a limitation. Furthermore, the model lacks an adaptive immune system and therefore cannot fully reproduce the complex host–pathogen interactions. Additionally, only three *S. aureus* and three *E. faecalis* clinical isolates were tested in this proof-of-concept study, which limits the generalizability of the findings.

Several studies have previously established *G. mellonella* as an experimental platform for implant-associated biofilm research. Campos-Silva *et al*., introduced an in-larva biofilm infection model using toothbrush bristles as surrogate implants and applied vancomycin at 50 mg/kg as a positive control, demonstrating feasibility of antibiotic testing but without clinical pharmacokinetic rationale^20^. Mannala *et al.*, further adapted the model to stainless-steel and titanium implants, confirming biofilm formation and host interaction on metallic surfaces^21^. Zhao et al., recently evaluated antibiotic-loaded bone cement implants in *G. mellonella*, integrating survival analysis, CFU quantification, and SEM imaging of explanted material^22^. Collectively, these studies validated *G. mellonella* as a cost-effective in vivo model to study device-associated infections while promoting the 3R principles.

Our study expands upon these models in several essential aspects. First, we employed expanded ePTFE, a clinically approved cardiovascular suture material routinely used in valve reconstruction and vascular surgery, thereby increasing the translational relevance compared with previously used non-clinical substrates. Second, we performed a direct comparison of two infection routes—biofilm formation inside larvae (IL) and transplantation of pre-formed biofilms (PF), allowing the differentiation of host-mediated effects from biofilm maturation processes. Third, we integrated survival, quantitative CFU analysis, and SEM imaging in the same experimental system, providing a comprehensive readout that has not been reported together in prior implant studies. Finally, we applied clinically relevant antibiotic concentrations derived from human peak plasma levels rather than conventional high-dose regimens such as 50 mg/kg vancomycin commonly used in previous models. This adjustment enhances the physiological relevance of treatment evaluation and bridges the translational gap between invertebrate and mammalian studies. This approach enhances the translational potential of the *G*. *mellonella* model, offering a cost-effective, ethical, and 3Rs-aligned platform that serves as a scalable pre-screening tool before proceeding to vertebrate models in preclinical biofilm research.

## Materials and methods

### Bacterial isolates and implant material

Clinical isolates of *S. aureus* (SA14073, SA29552, SA31685) and *E. faecalis* (EF67230, EF1653, EF9367) (see Supplementary Tables S1 and S2) were obtained from the Institute of Medical Microbiology, Jena University Hospital, Germany. These isolates originated from diverse clinical infections, including endocarditis (SA14073, EF67230), bloodstream infections (SA29552, EF1653, EF9367), and wound infections (SA31685). Antimicrobial susceptibility was determined using the VITEK 2 automated system (bioMérieux, Marcy-l’Étoile, France) and interpreted following European Committee of Antimicrobial Susceptibility Testing (EUCAST) 2022 clinical breakpoints. *S. aureus* strains were cultivated in Mueller–Hinton broth (Oxoid Deutschland GmbH, Wesel, Germany), and *E. faecalis* strains in Todd–Hewitt broth (Oxoid Deutschland GmbH, Wesel, Germany). Bacteria were grown at 37 °C under shaking conditions until reaching early exponential growth and adjusted to a 0.5 McFarland standard (~ 10⁸ CFU/mL) using a Multiscan GO spectrophotometer (Thermo Fisher Scientific, USA). This standardized inoculum served as the working suspension for subsequent experiments.

For this study, we employed ePTFE cardiovascular suture (W. L. Gore & Associates GmbH, Putzbrunn, Germany) as a surrogate implant. The ‘expanded’ structure refers to the microporous architecture produced during manufacturing, which enhances biocompatibility and tissue integration, critical factors in cardiovascular applications^23^. ePTFE is a clinically approved biomaterial widely used in cardiovascular and valve reconstruction surgeries, including annuloplasty and prosthetic valve replacement, where it is favored over alternatives such as Dacron due to its microporous structure, high biocompatibility, and reduced thrombogenicity^24^. These characteristics make ePTFE particularly relevant for modeling implant-associated endocarditis, as biofilm formation often initiates at the suture–tissue interface. Using a clinically validated material therefore increases the translational relevance of the *G. mellonella* implant model. For the biofilm experiments, ePTFE suture was cut to 0.5 cm in length (as it was already 0.2 mm in diameter) using a sterile scalpel for in vitro and in vivo implant biofilm.

### in vitro biofilm formation

Early log-phase bacterial suspensions (0.5 McFarland) were used to inoculate sterile 96-well microplates (200 µL per well, triplicates per strain). The ePTFE were incubated at 37 °C in a shaking incubator at 160 RPM in the bacterial suspension in the microtiter plate. The medium was refreshed after 24 h of incubation. After 48 h, bacterial suspension was removed, and the implants were washed twice with 200 µL of 1 x PBS to remove unattached bacteria, sonicated for 15 min^25^, vortexed for 30 s, serially diluted, plated on MH agar plates, and CFU was counted after 24 h of incubation at 37 °C. The experiments were performed with three technical replicates and three biological replicates (*n* = 3 × 3 = 9).

### *G. mellonella* systemic infection model

*G. mellonella* wax moth larvae were obtained from Bruno Mariani-FLOTEX (Augsburg, Germany). Final instar larvae were stored in a refrigerator at 15 °C for a maximum of 14 days and all experiments with the larvae were performed within this time period. In the experiments, larvae of an average size of 3.3 cm (with a standard deviation (SD) of ± 0.12 cm) were used.

For infection induction, bacterial cultures in the early logarithmic growth phase were centrifuged at 3000 × *g* for 10 min, and the pellets were resuspended in sterile 1× PBS to a final concentration of 10^9^ CFU/mL. Serial dilutions were prepared, and 10 µL of suspensions containing 10^7^, 10^8^, or 10^9^ CFU/mL were injected into the proleg of *G. mellonella* larvae using a Hamilton syringe (Hamilton Bonaduz AG, Bonaduz, Switzerland), corresponding to infection doses of 10^5^, 10^6^, and 10^7^ CFU per larva, respectively. Larval survival was recorded at 24 h intervals for up to 72 h, and survival data were analyzed using Kaplan–Meier plots. Larval survival was monitored using the pathology scoring index^13^; larvae that were fully melanized, immobile, and showed no response to touch were considered dead. Each experiment contained five larvae per bacterial dose and was repeated thrice (*n* = 5 × 3 = 15), including mock treatment (1 x PBS injection).

### in vivo biofilm formation - establishing implant-associated infections within the larvae

**Fig. 8:**
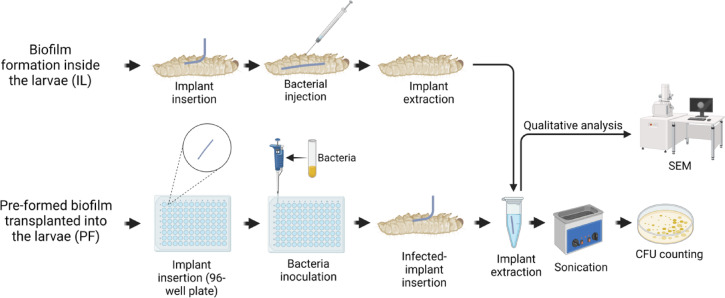
Schematic representation of the biofilm formation approaches and experimental workflow. IL: biofilm development on implants within larvae (infection established in vivo); PF: implantation of pre-formed in vitro biofilms into larvae. Larval survival was monitored for 48 h post-implantation, after which implants were retrieved for CFU enumeration and microscopic examination.

Two experimental approaches were used to establish implant-associated biofilms: (i) inside the larva (IL), biofilm formation on the implant inside the larvae reflecting hematogenous spreading of pathogens and (ii) pre-formed biofilm implantation (PF), in which biofilms were cultivated on the implant prior to insertion into the larvae (Fig. 8).

In the IL group (*n* = 5 × 3 = 15), sterile implants were inserted into the larvae, and after 1 h, infection was induced by injecting 10^5^ CFU/larva through the proleg. In the PF group (*n* = 5 × 3 = 15), ePTFE implants were pre-incubated with bacterial suspensions adjusted to 0.5 McFarland in 96-well microplates for 24 h at 37 °C under shaking conditions (160 rpm), followed by implantation into the larvae via the proleg. A control group (*n* = 5 × 3 = 15) received sterile implants and 10 µL of sterile 1× PBS, serving as non-infected implant-only controls. All larvae were incubated for 48 h post-implantation. Survival was monitored throughout the experiment, and for bacterial quantification, implants were removed, washed, and processed for CFU enumeration as described above. Selective media were used for differential cultivation: mannitol salt agar (MSA; Roth, Karlsruhe, Germany) for *S. aureus* and Todd–Hewitt agar supplemented with 8 mg/L tetracycline (AppliChem GmbH, Darmstadt, Germany) for *E. faecalis*, thereby suppressing the larvae’s endogenous enterococcal microbiota. Freshly prepared media and antibiotics were used in all experiments.

### Scanning electron microscopy (SEM)

Biofilm-coated ePTFE implants (from in vitro and in vivo experiments) were fixed in modified Karnovsky’s fixative (2.5% glutaraldehyde, 4% paraformaldehyde in 0.1 M sodium cacodylate buffer, pH 7.4) for 1 h at room temperature. Samples were rinsed three times (15 min each) in the same buffer, dehydrated through graded ethanol (30–100%), and critical-point dried using liquid CO₂. Gold sputter-coating (~ 2 nm) was performed using a CCU-010 coater (Safematic GmbH, Switzerland), and imaging was carried out with a field-emission SEM LEO-1530 Gemini (Carl Zeiss NTS GmbH, Germany). Three independent replicates per strain were analyzed.

### Antimicrobial treatments in the larvae

To compare the antibiotic biofilm eradicating efficacy between the IL and PF routes of larval implant infection, larvae were treated with single and combined doses of vancomycin and rifampicin. For the IL experiment, larvae were inserted with sterile implants and post 1 h implantation, infected with 10^5^ CFU/larvae bacterial dose and incubated for 48 h at 37 °C. Post 48 h incubation, alive larvae were treated with either 20 mg/L of vancomycin, 5 mg/L of rifampicin, or a combination of 20 mg/L vancomycin and 5 mg/L rifampicin (both Sigma-Aldrich Chemie GmbH, Taufkirchen), administered via a Hamilton syringe through a different proleg to prevent hemolymph loss. For the PF experiment, the implants were incubated in a bacterial suspension adjusted to 0.5 McFarland for 24 h and then implanted into the larvae and incubated for 24 h. Post incubation, larvae were treated with same antibiotic concentrations. For consistency, bacterial inoculation was always performed in the second right proleg. Antibiotic treatments were subsequently administered in the third right proleg, directly below the inoculation site. This standardized approach minimized variability, ensured uniform drug distribution into the hemolymph, and avoided repeated puncture of the same proleg, which could otherwise lead to hemolymph leakage. The concentrations were selected based on human blood peak plasma concentration of vancomycin^19^ and rifampicin^26^. The antibiotic concentrations were chosen to approximate human therapeutic exposures. Vancomycin typically achieves peak plasma concentrations of ~ 20–40 mg/L after intravenous dosing in patients. Accordingly, we selected 20 mg/L as a conservative surrogate concentration. Similarly, rifampicin achieves peak plasma concentrations of ~ 5–10 mg/L in humans after standard oral or intravenous dosing. Therefore, 5 mg/L was chosen to reflect a clinically relevant exposure within this range. The dosage was adjusted based on larvae hemolymph average volume of ~ 50 µL^13^. Larvae were monitored for survival at 24 h post-treatment by assessing melanization, movement, and response to touch. Mortality was recorded as non-responsive larvae with complete melanization^13^. Each experimental group included five larvae per isolate and treatment condition, and all experiments were independently repeated thrice (*n* = 5 × 3 = 15). After 24 h of treatment, alive larvae were surgically opened, and the implants were carefully removed. The implants were rinsed with 1x PBS, sonicated, vortexed and subsequently plated for CFU enumeration as described above.

## Statistical analysis

Data were analyzed using GraphPad Prism 9 (GraphPad Software Inc., USA). Survival analysis were assessed using the Log-rank (Mantel–Cox) test, while CFU data were evaluated with the Kruskal–Wallis test. Significance was defined as *p* < 0.05.

## Supplementary Information

Below is the link to the electronic supplementary material.

Supplementary Material 1

## Data Availability

The data is available from the corresponding author upon request.

## Abbreviations

CFU: Colony-forming unit
ePTFE: Expanded polytetrafluoroethylene
*G. mellonella*: *Galleria mellonella*
IL: Implant-in-larvae model
PF: Pre-formed biofilm model
PBS: Phosphate-buffered saline
MH: Mueller-Hinton
SEM: Scanning electron microscopy
MRSA: Methicillin-resistant *Staphylococcus aureus*
MSSA: Methicillin-susceptible *Staphylococcus aureus*
