# Supplementary material for: Implant-associated biofilms of Staphylococcus aureus and Enterococcus faecalis clinical isolates on expanded polytetrafluoroethylene suture in Galleria mellonella model
Source: Sci Rep. 2025 Nov 12;15:39555. doi: 10.1038/s41598-025-26971-5 (PMC12612210; doi:10.1038/s41598-025-26971-5)
Supplement: Supplementary file 1 — Supplementary Material 1 [file 41598_2025_26971_MOESM1_ESM.docx]

# Supplementary Data

**Table S1** Antibiogram of *S. aureus* strains used in this study.

|  | ***S. aureus*** | | |
| --- | --- | --- | --- |
| **Strain** | **SA14073** | **SA29552** | **SA31685** |
| **Specimen** | Blood culture | Catheter material | Wound swab |
| **Clinical background** | Endocarditis | Catheter-related blood-stream infection | Wound infection |
| Macrolide | 8 (R) | 8 (R) | 0.25 (S) |
| Clindamycin | 8 (R) | 8 (R) | 0.25 (S) |
| Vancomycin | 1 (S) | 2 (S) | 1 (S) |
| Penicillin | 0.5 (R) | 0.5 (R) | 0.5 (R) |
| Flucloxacillin | 4 (R) | 4 (R) | 0.25 (S) |
| Linezolid | 2 (S) | 2 (S) | 2 (S) |
| Rifampicin | 0.5 (S) | 0.5 (S) | 0.5 (S) |
| Daptomycin | 0.25 (S) | 2 (R) | 0.25 (S) |
| Fosfomycin | 8 (S) | 8 (S) | 8 (S) |
| Ciprofloxacin | 8 (R) | 8 (R) | 0.5 (S) |
| Moxifloxacin | 4 (R) | 8 (R) | 0.25 (S) |
| Gentamicin | 0.5 (S) | 0.5 (S) | 0.5 (S) |
| Tigecycline | 0.12 (S) | 0.12 (S) | 0.12 (S) |
| Profile | MRSA | MRSA | MSSA |

* MRSA (methicillin-resistant *Staphylococcus aureus*), MSSA (methicillin susceptible *Staphylococcus aureus*), S (sensitive), R (resistant)

**Table S2** Antibiogram of *E. faecalis* strains used in this study.

|  | ***E. faecalis*** | | |
| --- | --- | --- | --- |
| **Strain** | **EF67230** | **EF1653** | **EF9367** |
| **Specimen** | Mitral valve swab | Blood culture | Blood culture |
| **Clinical background** | Endocarditis | Urosepsis | Recurrent bacteremia |
| Vancomycin | 2 (S) | 1 (S) | 1 (S) |
| Teicoplanin | <0.5 (S) | < 0.5 (S) | <0.5 (S) |
| Linezolid | 2 (S) | 2 (S) | 2 (S) |
| Amoxicillin | <2 (S) | <2 (S) | 2 (S) |
| Ampicillin | <2 (S) | <2 (S) | 2 (S) |
| Moxifloxacin | 0.5 (S) | 0.5 (S) | 1 (S) |
| Levofloxacin | 2 (I) | <10 (R) | 2 (I) |
| Tigecycline | <0.12 S | <0.12 (S) | <0.12 (S) |

S (sensitive), R (resistant)


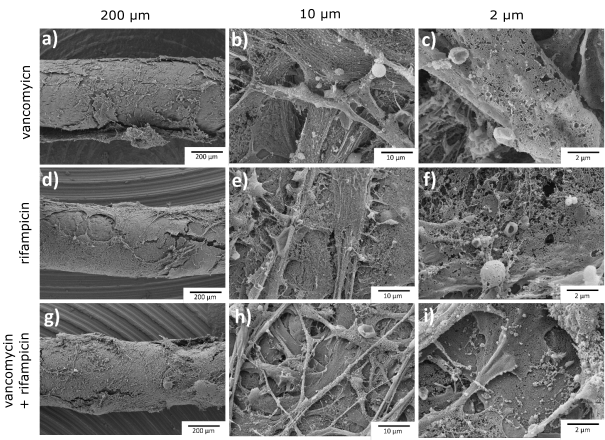


Figure S1 The post antibiotic treatment SEM analysis of *S. aureus* SA14073 biofilms formed on implants with PF methodology. The biofilm treated with 20 mg/L vancomycin (a-c), 5 mg/L rifampicin (d-f) and combination of vancomycin and rifampicin (g-i). The larvae were injected with *S. aureus* SA14073 pre-infected implant and incubated, post 48 h incubation, larvae were treated with antibiotics. After 24 h of treatment, implants were taken out of the larvae, washed with 1 x PBS, fixed and SEM was performed. The images showed different magnifications as indicated on the panels. The scale bar (black) represents 200 µm (a, d, g), 10 µm (b, e, h) and 2 µm (c, f, i).
